# Development and Validation of a Stratification Tool for Predicting Risk of Deep Sternal Wound Infection after Coronary Artery Bypass Grafting at a Brazilian Hospital

**DOI:** 10.21470/1678-9741-2016-0030

**Published:** 2017

**Authors:** Michel Pompeu Barros Oliveira Sá, Paulo Ernando Ferraz, Artur Freire Soares, Rodrigo Gusmão Albuquerque Miranda, Mayara Lopes Araújo, Frederico Vasconcelos Silva, Ricardo de Carvalho Lima

**Affiliations:** 1 Division of Cardiovascular Surgery, Pronto-Socorro Cardiológico de Pernambuco (PROCAPE), Recife, PE, Brazil.

**Keywords:** Coronary Artery Bypass, Wound Infection, Risk Assessment/Methods

## Abstract

**Objective:**

Deep sternal wound infection following coronary artery bypass grafting is a serious complication associated with significant morbidity and mortality. Despite the substantial impact of deep sternal wound infection, there is a lack of specific risk stratification tools to predict this complication after coronary artery bypass grafting. This study was undertaken to develop a specific prognostic scoring system for the development of deep sternal wound infection that could risk-stratify patients undergoing coronary artery bypass grafting and be applied right after the surgical procedure.

**Methods:**

Between March 2007 and August 2016, continuous, prospective surveillance data on deep sternal wound infection and a set of 27 variables of 1500 patients were collected. Using binary logistic regression analysis, we identified independent predictors of deep sternal wound infection. Initially we developed a predictive model in a subset of 500 patients. Dataset was expanded to other 1000 consecutive cases and a final model and risk score were derived. Calibration of the scores was performed using the Hosmer-Lemeshow test.

**Results:**

The model had area under Receiver Operating Characteristic (ROC) curve of 0.729 (0.821 for preliminary dataset). Baseline risk score incorporated independent predictors of deep sternal wound infection: obesity (*P*=0.046; OR 2.58; 95% CI 1.11-6.68), diabetes (*P*=0.046; OR 2.61; 95% CI 1.12-6.63), smoking (*P*=0.008; OR 2.10; 95% CI 1.12-4.67), pedicled internal thoracic artery (*P*=0.012; OR 5.11; 95% CI 1.42-18.40), and on-pump coronary artery bypass grafting (*P*=0.042; OR 2.20; 95% CI 1.13-5.81). A risk stratification system was, then, developed.

**Conclusion:**

This tool effectively predicts deep sternal wound infection risk at our center and may help with risk stratification in relation to public reporting and targeted prevention strategies in patients undergoing coronary artery bypass grafting.

**Table:** 

Abbreviations, acronyms & symbols	
aROC BMI BHIS CABG CDC COPD CPB DSWI ITA OR ROC	= Area of receiver operating characteristic curve = Body mass index = Brompton & Harefield Infection Score = Coronary artery bypass grafting = Centers for Disease Control and Prevention = Chronic obstructive pulmonary disease = Cardiopulmonary bypass = Deep sternal wound infection = Internal thoracic artery = Operation room = Receiver operating characteristic

## INTRODUCTION

Deep sternal wound infection (DSWI) following coronary artery bypass grafting (CABG) is a serious and costly complication^[1]^. Although individual risk factors for DSWI after CABG have been identified in multiple previous studies^[2-6]^, and despite the existence of stratification tools for predicting risk of surgical site infection after CABG [for instance, the Brompton & Harefield Infection Score (BHIS) developed by Raja et al.^[7]^, which included leg or sternal, superficial, deep incisional, or organ/space surgical site infections], there is a lack of specific risk stratification tools to predict DSWI after CABG.

This study was undertaken to develop a specific prognostic scoring system for the development of DSWI that could risk-stratify patients undergoing CABG and should be applied right after the end of the surgical procedure.

## METHODS

### Study Design

The study was conducted in accordance with the principles of the Declaration of Helsinki. The local ethical committee approved the study. The authors adhered to STROBE guidelines^[8]^ for reporting observational studies.

Continuous, prospective surveillance data on DSWI was collected. From March 2007 to August 2016, for every CABG (with or without additional procedure), a set of 27 variables were collected to allow subsequent analysis at our institution. The dependent variable was DSWI after surgical procedure. This variable was categorized into yes or no. DSWI was considered in those who met the criteria according to the Centers for Disease Control and Prevention (CDC)^[9]^:

1. Patient has organisms cultured from sternal/mediastinal tissue or fluid obtained during a surgical operation or needle aspiration;
2. Patient has evidence of mediastinitis seen during a surgical operation or histopathologic examination;
3. Patient has at least one of the following signs or symptoms with no other recognized cause: fever (38ºC), chest pain, or sternal instability and at least one of the following:
  - purulent discharge from sternal/mediastinal area;
  - organisms cultured from blood or discharge from sternal/ mediastinal area;
  - mediastinal widening on X-ray.

The independent variables were:

- Age > 70 years;
- Gender (male or female);
- Obesity (body mass index - BMI ≥ 30 kg/m^2^);
- Hypertension (reported by patient and/or use of anti-hypertensive medication);
- Diabetes (reported by patient and/or use of oral hypoglycemic medication and/or insulin);
- Smoking (reported by patient; active or inactive for less than 10 years);
- Chronic obstructive pulmonary disease - COPD (dyspnea or chronic cough and prolonged use of bronchodilators or corticosteroids and/or compatible radiological changes - hypertransparency by hyperinflation and/or rectification of ribs and/or diaphragmatic rectification);
- Preoperative renal disease (creatinine ≥ 2.26 mg/dL or pre-operative dialysis);
- Previous cardiac surgery;
- Ejection fraction < 50%;
- Preoperative stay > 24h;
- Emergency surgery (during acute myocardial infarction, ischemia not responding to therapy with intravenous nitrates, cardiogenic shock);
- Use of internal thoracic arteries (ITA);
- Use of bilateral ITA;
- Harvesting technique for ITA (Pedicled - direct dissection of surrounding margin of tissue around the ITA with electrocautery - or Skeletonized - artery dissection with scissors and clipping intercostal branches with metal clips without involving any margins tissue around ITA);
- Number of bypasses;
- Use of cardiopulmonary bypass - CPB (on-pump or off-pump);
- Time of CPB > 100 minutes;
- Additional surgical procedure;
- First-year resident in the operation room (OR);
- Postoperative low cardiac output;
- Reoperation (new sternotomy for bleeding, tamponade, or other reasons during the intra-hospital period);
- Respiratory complications (pulmonary infection, acute respiratory distress syndrome, atelectasis, need for intubation for more than 48 hours);
- Postoperative renal complications (creatinine ≥ 2.26 mg/dL or postoperative dialysis);
- Blood transfusion (blood transfusion in the postoperative period before diagnostic definition of mediastinitis);
- Multiple transfusions (more than 3 units of any blood products in postoperative period before diagnostic definition of DSWI);
- Infection at another site.

### Data Analysis

Binary logistic regression analysis was performed to identify any independent predictors of DSWI in our population, with outcome measure of DSWI detected during primary admission or on readmission. Calibration of the scores was performed using the Hosmer-Lemeshow goodness-of-fit test. For the Hosmer-Lemeshow test, a *P* value that was not statistically significant (e.g, *P* greater than 0.05) was considered to indicate reasonable model fit. Discrimination power of the scores was analyzed using the receiver operating characteristic (ROC) curve.

Somers’ D_xy_ rank correlation coefficient was used as a measure of discrimination. D_xy_ corresponds to 2*(C-0.5) where C is the generalized area of ROC (aROC) curve (concordance probability).

R version 2.15.2 (http://www.R-project.org) and rms package (R package version 4.0-0, http://CRAN.R-project.org/package¼rms) statistical software package was used for statistical analysis.

## RESULTS

### Population

The total sample size of this study was 1500 cases. We initially developed a predictive model in a subset of 500 consecutive cases drawn from our hospital (March 2007-April 2010). Following testing of the preliminary data, the dataset was expanded to other 1000 consecutive cases (March 2010-August 2016), from the same hospital.

### Univariate Analysis

Variables that were associated with increased risk of DSWI with *P*<0.05 were obesity, diabetes, smoking, preoperative renal disease, COPD, ejection fraction < 50%, use of pedicled ITA, on-pump CABG, additional procedure to CABG, renal complications, respiratory complications, infection at another site, reoperation and multiple transfusions. Table 1 shows the data from the univariate analysis.

**Table 1:** Incidence of mediastinitis according to preoperative, intraoperative and postoperative variables (univariate analysis).

Variable	*P* value	Odds ratio (95% confidence interval)
Age > 70 years	1.000^(1)^	1.00 (0.41-2.41)
Male	0.237^(1)^	1.50 (0.66-3.38)
Obesity	0.014^(2)^	2.97 (1.29-6.84)
Hypertension	1.000^(2)^	1.01 (0.30-3.48)
Diabetes	0.004^(1)^	3.03 (1.37-6.71)
Smoking	0.011^(1)^	2.67 (1.22-5.83)
Renal disease	0.025^(2)^	3.21 (1.22-8.40)
COPD	< 0.001^(2)^	6.42 (2.76-14.96)
Previous cardiac surgery	0.196^(1)^	1.97 (0.71-5.41)
EF < 50%	0.036^(1)^	2.25 (1.03-4.89)
Preoperative stay > 24h	0.680^(1)^	0.80 (0.70-1.10)
Number of bypasses	0.648^(1)^	1.50 (0.62-3.63)
Use of ITA	0.306^(1)^	0.62 (0.24-1.67)
Bilateral ITA	0.071^(1)^	1.10 (0.20-1.90)
Pedicled ITA	0.004^(2)^	5.28 (1.53-18.21)
On-pump CABG	0.012^(1)^	2.92 (1.15-7.72)
CPB > 100 min	0.124^(1)^	2.08 (0.81-5.35)
Additional procedure	0.031^(1)^	5.54 (1.44-21.42)
Emergency surgery	0.371^(2)^	2.46 (0.38-11.78)
First-year resident in the OR	0.234^(2)^	1.11 (0.30-1.90)
Low cardiac output	0.426^(2)^	1.47 (0.58-3.74)
Renal complications	< 0.001^(2)^	7.51 (3.11-18.11)
Respiratory complications	0.001^(2)^	4.80 (2.10-10.97)
Infection at another site	< 0.001^(2)^	20.37 (8.19-51.21)
Reoperation	< 0.001^(1)^	82.4 (30.4-223.3)
Any blood transfusion	0.070^(1)^	2.21 (0.87-5.83)
Multiple transfusion	0.003^(1)^	3.33 (1.52-7.29)

CABG=coronary artery bypass graft; COPD=chronic obstructive pulmonary disease; CPB=cardiopulmonary bypass; EF=ejection fraction; ITA=internal thoracic artery; OR=operation room

* Significant difference at 5.0%;

(1) Chi-square test;

(2) Fisher’s exact test

### Multivariate Analysis by Logistic Regression

We identified the following independent risk factors for developing DSWI: obesity (*P*=0.046; OR 2.58; 95% CI 1.11-6.68), diabetes (*P*=0.046; OR 2.61; 95% CI 1.12-6.63), smoking (*P*=0.008; OR 2.10; 95% CI 1.12-4.67), pedicled ITA (*P*=0.012; OR 5.11; 95% CI 1.42-18.40), and on-pump CABG (*P*=0.042; OR 2.20; 95% CI 1.13-5.81).

### Predictive Model

**Fig. 1:**
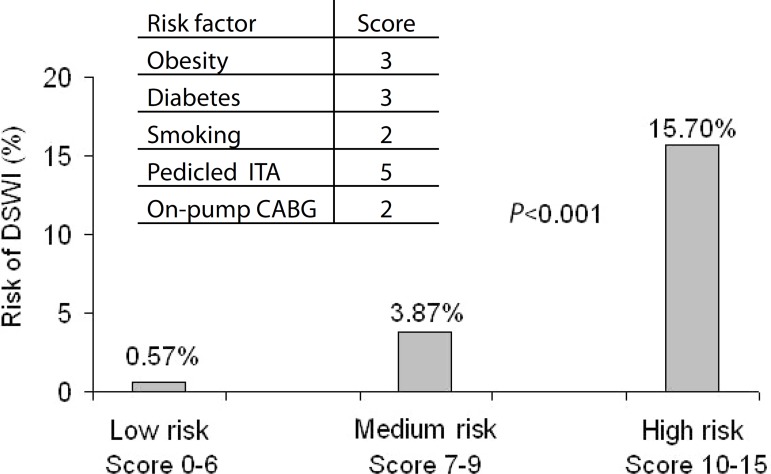
Tool to predict DSWI in patients undergoing CABG.

The score was devised by rounding off the OR values in the multivariate logistic regression, assigning 3 points to obesity, 3 points to diabetes, 2 points to smoking, 5 points to pedicled ITA, and 2 points to on-pump CABG (Figure 1).

**Fig. 2:**
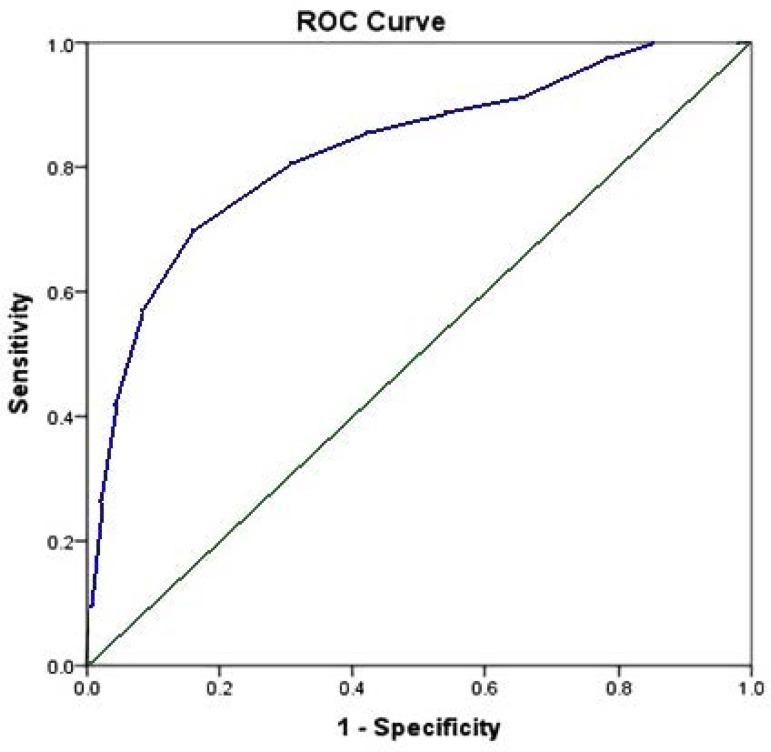
Receiver operating characteristic (ROC) curve for initial predictive model.

The initial predictive model in a subset of 500 cases offered a very good prediction of outcome. The aROC curve was 0.821 (Figure 2). The Hosmer-Lemeshow goodness-of-fit test (chi significance) showed a score of 0.983.

**Fig. 3:**
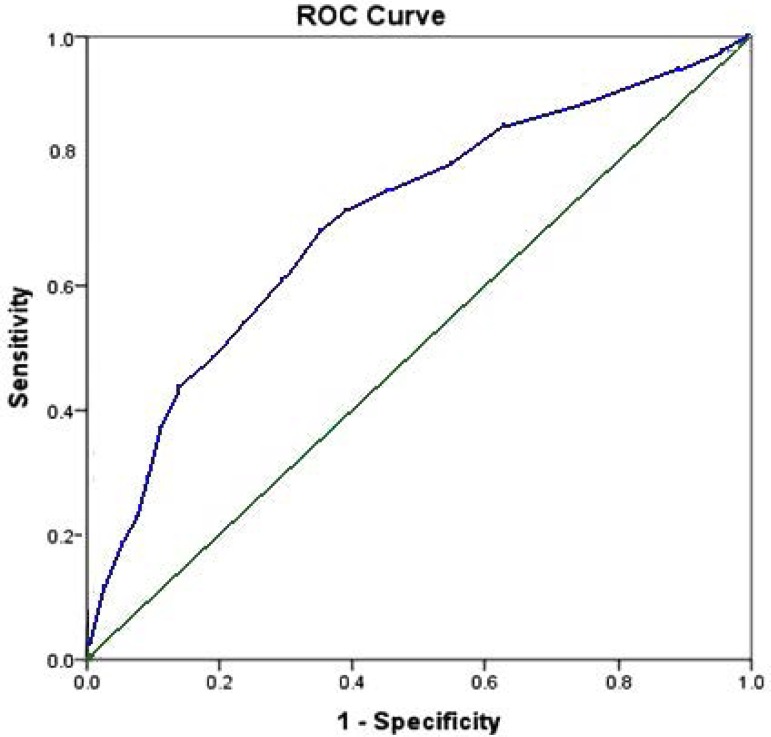
Receiver operating characteristic (ROC) curve for final predictive model.

The predictive model was tested and found to predict outcome effectively in the larger dataset (aROC curve was 0.729) (Figure 3). Hosmer-Lemeshow test showed a score of 0.142. Bootstrapping validation confirmed a good discriminative power of the model (preliminary dataset D_xy_=0.61, testing dataset D_xy_=0.42).

## DISCUSSION

This study was undertaken at a tertiary care hospital that perform large volumes of CABG surgery, and data from 1500 patients were used to analyze risk factors for DSWI after CABG surgery. Obesity, diabetes, smoking, pedicled ITA and on-pump CABG were identified as specific predictors of DSWI after CABG. The present index was developed and validated as a predictive tool to specifically stratify CABG patients into three groups based on the risk of postoperative DSWI.

Many factors have been associated with the development of DSWI after cardiac surgery^[10]^. However, there is no consensus as to which factors are most important and how each one is an independent predictor of risk for postoperative DSWI^[10]^.

We observed obesity as an independent risk factor for postoperative DSWI. Milano et al.^[11]^ discussed some factors that could explain why obesity is a risk factor, for example, the dose of prophylactic antibiotics not corrected for BMI of the patient. They also suggest that skin preparation can be difficult and inappropriate due to the deep folds of the skin and fatty tissue itself, which can act as a substrate for infection. Diez et al.^[12]^ related the etiology of DSWI in obese patients with bradytrophic properties of adipose tissue that contribute to poor healing of wounds. Farsky et al.^[13]^ also found a BMI > 40 kg/m^2^ to be an independent predictor of DSWI after CABG among 1975 Brazilian patients.

Diabetes is always a feared risk factor and viewed with caution by cardiovascular surgeons, because, as a result of its pathophysiology, microvascular changes and high levels of blood glucose may adversely affect the healing process^[14,15]^. In this study, diabetic patients were 2.6 times more likely (independent association) to develop DSWI compared with non-diabetics. Despite our results, in another Brazilian study, published by Tiveron et al.^[16]^, diabetes was not found to be independently associated with mediastinitis among 2768 patients when it was adjusted for renal disease. On the other hand, Ledur et al.^[17]^ found diabetes to be independently associated with any infection after CABG (including DSWI) among 717 Brazilian patients.Another independent risk factor for DSWI in our study was smoking, being associated with 2.1 times more likely to present with DSWI compared with non-smokers. Abboud et al.^[18]^ also reported that smokers were 3.3 times more likely (independent association) to develop DSWI when compared with non-smokers in a case control study involving 117 patients (39 cases and 78 controls).

Our study found that on-pump CABG was an independent risk factor for developing postoperative DSWI. Bottio et al.^[19]^, in a prospective study with 324 patients who underwent CABG, of whom 216 underwent on-pump CABG and 108 underwent off-pump, observed there was lower incidence of DSWI in the off-pump group, although this difference was not statistically significant. Mack et al.^[20]^ observed a lower incidence of wound infection in patients undergoing off-pump compared to on-pump. Sabik et al.^[21]^, in the Cleveland Clinic study involving 812 patients undergoing CABG (half on-pump and half off-pump), have identified a higher incidence of wound infection in the on-pump group (2% *vs.* 0.2%, *P*=0.04). Reston et al.^[22]^, in a meta-analysis of 53 studies involving a total of 46621 patients, found lower incidence of wound complications, including DSWI, in patients undergoing off-pump compared with on-pump CABG.

We did not observe any differences in the incidence of DSWI among patients who used or not ITA and nor did we observe an increased risk for those who used bilateral ITA. However, we found that there was a higher incidence of DSWI in patients who used pedicled ITA compared with skeletonized ITA (statistically significant). In other words, the skeletonized ITA was a protective factor for postoperative DSWI, which was an independent association.

Several studies have shown favorable results to the use of skeletonized ITA^[23-26]^. Saso et al.^[23]^ demonstrated that skeletonization of ITA in patients undergoing CABG was associated with reduced incidence of DSWI (OR 0.41, 95% CI 0.26 to 0.64) and this effect was even more evident when the specific analysis of diabetic patients was performed (OR 0.19, 95% CI from 0.1 to 0.34). Kai et al.^[24]^ observed that the incidence of DSWI was significantly lower in the group that underwent CABG with use of skeletonized ITA compared to the group using pedicled ITA (0.6% *vs.* 13% *P*=0.01). Sá et al.^[25]^ performed a meta-analysis with 4817 patients from 22 studies, demonstrating that skeletonized ITA appears to reduce the incidence of postoperative DSWI in comparison to pedicled ITA after CABG. Sá et al.^[26]^ conducted a second meta-analysis to determine whether there was any difference between skeletonized *versus* pedicled bilateral ITA in terms of DSWI after CABG with 8 studies involving 2633 (1698 skeletonized; 935 pedicled) and concluded that, when both ITAs are used, the skeletonized technique appeared to reduce the incidence of DSWI after CABG in comparison to the pedicled technique.

These results were found probably as a result of better sternal perfusion after ITA skeletonization compared to the pedicled ITA^[27,28]^. Boodhwani et al.^[27]^ conducted a study with 48 patients, in which each individual was submitted to CABG using bilateral ITA, and all ITAs were dissected skeletonized in left side and pedicled in right side. Patients were then evaluated for sternal perfusion through scintigraphy (radionuclear image). The authors found that sternal perfusion was increased in skeletonized side compared with pedicle side (increase of 17.6%, *P*=0.03). Kamiya et al.^[28]^ showed that the oxygen saturation and blood flow in the microcirculation of the sternum tissue were better when using the skeletonized ITA compared to pedicled ITA. Despite the beneficial impact of skeletonization on reducing the risk of sternal wound infection it is important to emphasize that skeletonization is technically more demanding and more time-consuming than pedicled ITA harvesting with a steep learning curve associated with it^[29-31]^. Furthermore, some surgeons have raised concerns about the quality of the graft, mainly when it comes to the patency and the flow capacity of the skeletonized ITA. According to two meta-analyses^[32,33]^, these concerns may be unfounded. The first one^[32]^ was conducted in order to determine whether there was any difference between skeletonized *versus* pedicled ITA in terms of patency within the first two years after CABG. In this meta-analysis, five studies involving 1764 evaluated conduits (1145 skeletonized; 619 pedicled) met the eligibility criteria. The overall OR for graft occlusion showed no statistical significant difference between groups. The authors concluded that, in terms of patency, skeletonized ITA appears to be non-inferior in comparison to pedicled ITA after CABG.

The second one^[33]^ aimed to summarize the evidence comparing the free flow capacity of skeletonized *versus* pedicled ITA during CABG. In total, 8 studies were identified and involved a total of 907 conduits (360 skeletonized and 547 pedicled). The authors concluded that, in terms of flow capacity, the skeletonized ITA appears to be superior in comparison to pedicled ITA during CABG.

One of the novelties of this risk prediction score, compared to other existing scores, is that it was designed to be applied not in the preoperative period, but at the time the surgical procedure ends, so that we have a score based not only on preoperative factors, but also on what actually happened during the surgical procedure.

Our study has several potential limitations. Firstly, other risk factors may be involved, but they are difficult to be measured. The aspect of the bone, which can sometimes show signs of osteoporosis, ischemia, the surgeon’s ability, failure to follow the antisepsis procedures, errors in the sternotomy and in the sternum rewiring, and excessive use of an electric scalpel, are factors that are very often not mentioned, but can be important factors in the pathophysiology of DSWI. Secondly, the total number of DSWI events was relatively small (n=72), limiting the ability to identify associations with a large number of variables. In addition, as the study emanates from one centre, one could argue that it is limited in its ability to identify associations between other unrecognized risk factors and DSWI. Similarly, the accuracy (discrimination) and utility of this tool has been validated internally; however, its generalizability to other CABG practices is unknown. External validation by other institutions of these data is required to overcome these limitations. Despite these limitations, this tool was developed and validated as an accurate tool for predicting DSWI in CABG patients. This tool was able to discriminate between three different risk strata of patients using objective data.

## CONCLUSION

In conclusion, our results support the use of this tool for stratifying CABG patients based on risk of DSWI at our center. Given the wide DSWI risk variability among CABG patients, the practicing surgeon will be able to identify those at highest risk, providing the opportunity for postoperative planning, care and implementation of more aggressive preventive strategies when indicated.

**Table:** 

Authors’ roles & responsibilities	
MPBOS	Conception and design; operations and/or experiments performance; analysis and/or interpretation of data; statistical analysis; manuscript writing or critical review of its content; final approval of the manuscript
PEF	Conception and design; operations and/or experiments performance; analysis and/or interpretation of data; statistical analysis; manuscript writing or critical review of its content; final approval of the manuscript
AFS	Operations and/or experiments performance; manuscript writing or critical review of its content; final approval of the manuscript
RGAM	Operations and/or experiments performance; manuscript writing or critical review of its content; final approval of the manuscript
MLA	Operations and/or experiments performance; manuscript writing or critical review of its content; final approval of the manuscript
FVS	Manuscript writing or critical review of its content; final approval of the manuscript
RCL	Manuscript writing or critical review of its content; final approval of the manuscript
